# The politics of social-ecological justice and the paradox of economization

**DOI:** 10.1177/10245294251375504

**Published:** 2025-09-03

**Authors:** Francesco Laruffa

**Affiliations:** 1 27212 Department of Sociology, University of Geneva, Switzerland

**Keywords:** capitalism, economization, paradox, social-ecological justice, social-ecological transformation

## Abstract

This article highlights a central paradox of the current conjuncture: while capitalism is the root-cause of the current polycrisis, promoting social-ecological goals within this system possibly requires to *reinforce* some of its key features – and especially economization (i.e. the extension of profit-making logics to non-economic domains). In contemporary dominant strategies (policies for inclusive-green growth; ethical-political consumption; and Corporate Social Responsibility), solutions to social-ecological problems become investment objects that deliver growth and profits. While these strategies, if fully implemented, may potentially improve social-ecological outcomes, they would make the ideal of social-ecological justice more difficult to achieve. This problem of ‘non-linearity’ in social change generates an ‘unrealistic-pragmatism’ versus ‘necessary-utopianism’ dilemma for progressive forces: seemingly realistic strategies are actually unable to address the polycrisis whereas approaches discarded as utopian offer the only rational solution to it, namely abandoning the myth of endless growth and subordinating the economy to social-ecological needs.

## Introduction

This article contributes to debates on the possibility of moving to a ‘green economy’ and on the challenges related to making the green transition socially just. The concept of green economy is used in different ways. The most significant dividing line in the discussion is between those who see an environmentally sustainable economy as being compatible with profit-driven capitalism and those who reject this win-win scenario, assuming that building a green economy requires to move beyond growth-centred approaches (e.g. Jones and Ström, 2024). Thus, the discourse of the green economy ranges from a pro-growth approach that considers the greening of the economy as an investment opportunity and as a way to create more jobs, to a radical socioeconomic transformation oriented towards the achievement of a steady-state economy and degrowth (O’Neill and Gibbs, 2016: 1730). While the approach based on ‘green growth’ currently dominates national and international policy agendas, many scholars argue that, in order to promote social justice and human wellbeing in the respect of ecological limits, it is necessary to move beyond growth (e.g. Hickel, 2022; Schmelzer et al., 2022). Similarly, the literature on ‘just transition’ highlights that promoting social-ecological justice requires to go beyond a narrow focus on the just distribution of income, resources or employment opportunities, for integrating the recognitional, procedural, and restorative dimensions of justice (e.g. Abram et al., 2022). Rather than a technocratic matter, where the terms of the debate are established a priori by the dominant groups, a just transition is a political project that requires the *democratization* of social relations: people should be given the real opportunity to be heard for envisioning and realizing possible alternative futures, challenging the system of recognition that informs the existing social order (e.g. Schwanen, 2021; Stevis and Felli, 2015; Velicu and Barca, 2020). Finally, social-ecological justice needs to be global in scale. For example, moving to low-carbon energy provision systems in the Global North is increasing the demand for minerals and metals extracted through mining activities in the Global South, which undermines social-ecological justice there (Bainton et al., 2021). Social-ecological justice instead requires to reject those strategies in which green transitions in some regions of the world are achieved at the expense of other regions (Ajl, 2021).

But how can we concretely advance social-ecological justice? How can we move from less to more transformative understandings of green economy? In this article, I discuss some dominant strategies to promote social-ecological justice. National governments and international organizations try to make the ecological transition ‘just’ and ‘inclusive’ through various social and economic policies; private companies commit to social-environmental standards through practices of Corporate Social Responsibility (CSR); and many individuals participate in forms of ethical/political consumption, such as buying fair-trade and organic products. How do these currently prevailing efforts to advance social-ecological justice relate to capitalism? Do they enable transformative change within/beyond it?

Many scholars argue that since capitalism is responsible of the social, ecological, and political crises that we are facing today, it should be overcome in order to tackle them (e.g. Fraser, 2022). In particular, capitalism is inextricably linked with ecological degradation (e.g. Foster, 2002; Hickel 2022; Moore, 2015). In this view, green capitalism is only an illusion: realizing social-ecological justice demands to embrace ecosocialism (e.g. Löwy, 2015). While I agree with these claims (Laruffa, 2025), in this article I highlight a fundamental paradox of the transition towards more just and sustainable societies: as long as we remain trapped *within* capitalism, some of the approaches that pragmatically *promote* social-ecological goals *reinforce* some of its key features, especially economization and de-democratization. In turn, this paradox generates a dilemma for those actors struggling for progressive social change. The problem – which I call the ‘non-linearity’ of progressive social change – is that pragmatic steps undertaken to promote social-ecological goals within the current system may, on the long-term, undermine the possibility for a more radical social-ecological transformation beyond capitalism. Indeed, if promoting social-ecological goals within capitalism requires to further reinforce the latter, pursuing this strategy will make it more difficult to overcome this system – which, however, appears an essential precondition for fully realizing social-ecological justice.

To be sure, the concept of capitalism is an abstraction and generalization, which obscures important aspects of contemporary societies. As Gibson-Graham and Dombroski (2020) argue, the economy is ‘diverse’ and ‘heterogeneous’ rather than ‘monolithic’: it entails very different practices – including non-capitalist ones – and different ways of organizing economic activities, that is, of labouring beyond waged labour (e.g. through care work performed within families); of transacting beyond market exchange (e.g. through gift relationships or barter); and of enterprising beyond the logic of profit-maximization realized by privately owned firms (e.g. through cooperatives or non-profit enterprises). Even capitalist actors have different motivations and some of them are genuinely committed to the realization of social-ecological goals.

Yet the current hegemony of capitalism cannot be ignored (e.g. Dempsey, 2016: 16; Kenis and Lievens, 2015) and the existence of non-capitalist relations does not contradict this dominance. The problem is not only that capitalism often free rides on non-capitalist practices, such as the care work mostly performed by women on an unpaid basis (Fraser, 2022). The risk is also that non-capitalist practices remain trapped in ‘societal niches’, where ‘like-minded’ people with ‘shared values’ build ‘eco-social communities’, impeding to build ‘strategic agency’ and broad alliances across society (Bärnthaler, 2024: 3). However, contrary to the assumption that new common senses spread from such niche milieus to society more broadly, achieving the broad-based consent necessary for a deeper social-ecological transformation requires to start from ‘the common sense of the societal mainstream, while seeking to transform it’ (Bärnthaler, 2024: 5).

Against this background, this article contributes to recent debates on how to move to a post-capitalist, post-growth world (e.g. Ajl, 2021; Bärnthaler, 2024; Hickel, 2022; Schmelzer et al., 2022), interrogating the extent to which strategies undertaken *within* capitalism may constitute steps towards a different type of economic system. Indeed, some practices implemented to make capitalism social and green may actually open the way to more radical transformations. For example, while the idea of ‘circular economy’ has been incorporated in the projects of dominant actors such as business leaders, the European Commission and various governments that have framed it mainly in pro-capitalist and pro-growth terms, it can also be combined with degrowth principles and visions (Nesterova and Buch-Hansen, 2023). Similarly, the expansion of the fair-trade sector or of organic agriculture may be part of a broader project of transformation. My argument here is that there may be no linear relationship between steps undertaken to make capitalism more socially and ecologically responsible and steps aimed at moving to a post-capitalist world. Without opposing the idea that different visions of ‘green economy’, ‘green entrepreneurship’ and ‘eco-social policies’ range from less to more transformative ones (O’Neill and Gibbs, 2016; Schulze Waltrup, 2025), I challenge the idea of a linear transformative continuum.

The reminder of this paper is structured in five sections. The first section describes the main features of ‘social-ecological capitalism’: a potential future scenario, where some of the currently prevailing strategies for promoting social-ecological goals – such as ethical-political forms of consumption and CSR – are further strengthened. The second section identifies a central paradox that is at the core of these strategies, namely that they promote social-ecological goals through the reinforcement of some fundamental features of capitalism, especially economization and de-democratization. The third and fourth sections reflect on the dilemmatic implications of this paradox for progressive forces. Finally, the conclusion suggests some possible ways out of these dilemmas.

## The rise of social-ecological capitalism

There are many theories and definitions of capitalism and I do not have the space here to provide a detailed discussion of this topic. I thus only highlight some key aspects of capitalism. Indeed, while capitalism can take very different forms, it seems that its ‘essential feature’ involves the ‘investment of money in order to make a profit’ (Fulcher, 2015: 12). Thus, Marx (2008: 93–107) famously emphasized the fact that, under capitalism, means and ends are inverted: Commodities (C) – and thus the satisfaction of human needs – are treated as a mere means for obtaining more Money (M) and thus sustaining the endless accumulation of profits (in Marx’s formula: M-C-M′, where M′ is bigger than M). Moreover, capitalism conquers previously non-capitalist social relations, subjecting them to the economic logic of profit-maximization through continuous processes of ‘primitive accumulation’ – a concept, which is important also for understanding the relationship between capitalism and (neo)colonialism (e.g. Patnaik and Patnaik, 2021).

On this basis, in this article, I argue that a central feature of capitalism is that of promoting the *economization* of non-economic domains. Economization involves two distinct but mutually reinforcing dimensions. The first dimension entails the extension of the economic logic to non-economic domains. Referring to the de-politicized/technocratic and ‘epistemic’ aspects of economization, this dimension implies that, for example, public policies are largely viewed in economic terms and assessed in light of economic criteria. The second dimension of economization is political-distributive. It implies that the extension of profit-making opportunities in previously non-commodified realms benefit capital owners, the financial elites and big corporations at the expense of society at large whose needs remain either entirely unmet or badly satisfied. These two dimensions are connected through a reinforcing circle, as the technocratic extension of economic logics to new domains potentially creates new sources of accumulation (Laruffa, 2022). Hence, the more social-ecological problems are re-framed within the economic logic and as economic objects (the epistemic dimension), the more also new markets and thus new profit-making opportunities are created in previously uncolonized domains (the distributive dimension). Moreover, both dimensions point to another essential feature of capitalism, namely the limitation of democratic power that goes with it. On the one hand, to the extent that political decisions are translated into economic problems (e.g. issues of cost/benefit analysis and efficiency calculations) to be solved through de-politicized means (epistemic dimension), they can be delegated to technocratic agencies and ‘experts’ – especially economists – removing them from the democratic discussion. On the other hand, increasing the power of economic and financial elites (the distributive dimension) undermines political equality and thus weakens democracy. Capitalism then entails the expansion of the ‘economic’ at the expense of the ‘political-democratic’ in a twofold sense. First, capitalism implies the technocratic economization of politics, increasing the number of social domains that can be governed on the basis of cost-benefit calculations. Second, capitalism involves the transfer of political power from the public-democratic to the private-economic sphere, whereby the ‘market’ partially substitutes democratic citizenship.

A further characteristic of capitalism is that it is highly adaptable. In particular, as Boltanski and Chiapello (2005) argue, capitalism evolves and stabilizes itself integrating in its way of functioning the various critiques that are addressed to it. Thus, the norms and values that inform capitalist critiques – such as the ecological critique – ironically become drivers of capitalist accumulation and legitimization. Through a Gramscian ‘passive revolution’, capitalist elites transform the ecological critique into an economic opportunity, which reinforces commodification, whereas actors who previously adopted a critical and oppositional stance ‘are tempted to become part of the project and exchange conflict for cooperation, contestation for consensual governance’ (Kenis and Lievens, 2015: 12–13; see also Wanner, 2015).

Hence, over the last decades there have been various attempts to make capitalism more ‘social’ (or ‘inclusive’) and ‘green’. These efforts involved an emphasis on the positive role of social policy; the prioritization of poverty-reduction development strategies; and the investment in green technologies and renewable energies. This progressive shift within capitalism is driven by the need to partially compensate the losers of neoliberal globalization with a view to (re)construct hegemony and build consensus as well as by the imperative to solve the dysfunctions generated by capitalism itself (e.g. the excessive exploitation of nature) that undermine profitability on the long-term.

Therefore, in the process of answering to its critiques and in responding to the crises that it generates, capitalism evolves and transforms itself, becoming in certain aspects more socially and environmentally attentive. The solution to social-ecological problems generated by capitalism thus entails, paradoxically, even more capitalism – albeit of a social and green type. Some of the most important forms that this social-ecological turn is currently taking include the shift towards ‘inclusive green growth’ and ‘sustainable development’ at the level of (international) public policy; the adoption of Corporate Social Responsibility (CSR) and Environmental, Social and Governance (ESG) principles at the level of private companies; and the emergence of ethical-political forms of consumption at the individual level. Moreover, all three forms are deeply connected. The adoption of CSR/ESG-principles by companies and the labelling of their products accordingly is what enables ethical-political forms of consumption and investment, informing individuals about the social-environmental standards behind their consumption and investment choices. In turn, the more production, consumption and investment behaviours refer to social-ecological values, the more the whole economic system moves away from models of economic growth based on exclusion and unsustainability towards inclusive green growth. Below I elaborate more on each of these phenomena.

### CSR and ethical-political consumption

Both at the level of individual consumption choices and at the level of companies’ production strategies, it is possible to observe a growing sensibility towards socially and environmentally responsible products. Thus, more and more people (and states, via public procurement initiatives) buy those products that fulfil social-environmental criteria while boycotting (refusing to buy) those products that do not meet such standards (e.g. Lorenzini, 2022; Wilkinson, 2007). Parallel to this growing demand for ethical goods on the side of consumers, private companies have increasingly adopted CSR-principles in their business strategies (Kinderman, 2012). Committing to some (often minimal) social-ecological standards is seen not only as a way of doing the right thing in a moral sense: embracing these practices also makes sense from an economic viewpoint. In this context, CSR often becomes a marketing strategy aimed at increasing the company reputation (Sum and Ngai, 2005). These processes are also linked to ‘ethical finance’, which allows individuals/institutions to invest in those companies that respect social-environmental standards. In all these cases, firms’ profit-making activities are portrayed as compatible with the broader interests of society (Cabrera and Williams, 2014).

Taken together, these moralization processes may involve the co-optation of political activism by corporations. Through processes of ‘corporatization’, private companies come to dominate fields originally initiated by activists and social movements (King and Busa, 2017). Hence, these various forms of ethical production and consumption entail the commodification of political values, such as social justice and environmental sustainability. These values tend to be transformed – for example, through the labelling of products – into marketable commodities exchanged in ‘moralized markets’ (Balsiger, 2021; Suckert, 2018) – and this moralization of the economy also involves the reverse: the economization of morality (Sum and Ngai, 2005). Moralization processes can also be interpreted as a consequence of economization, whereby the locus of politics shifts from the democratic sphere to the market and socio-moral questions – traditionally the concerns of civic groups, democratic parliaments, trade unions and political parties – become ‘the business of market actors’ (Shamir, 2008: 3). Hence, the more social-ecological problems are treated as economic matters, the more societies need to appeal to voluntary moral commitments to promote social-ecological goals. Crucially, both the economic and the moral logics revolve around a ‘private’ rationality, marginalizing the political dimension on which democracy rests. Thus, the rise of ethical consumers and moral corporations entails the privatization of social-ecological concerns, as the state discharges its ‘responsibility to ensure equitable and ecologically sustainable means of social reproduction’ (Johnston, 2008: 262).

### Public policies

At the level of policymaking, the emergence of the inclusive green growth and sustainable development agendas entails the effort to combine the goals of social inclusion, economic growth and environmental sustainability in a coherent framework. In particular, the attempt to reconcile social and economic goals is visible in the emergence of ‘social investment’ as a social policy framework (Laruffa, 2022). Rather than viewing the welfare state as an economic cost, this approach suggests to re-frame social policies as investments that have not only social but also economic returns. The key argument is that many social policies positively contribute to economic growth, ‘competitiveness’ and sound public finances. For example, improving individuals’ health and education not only ameliorates their wellbeing but also enhances their ‘human capital’, facilitating their inclusion in the labour market and increasing their productivity. Similarly, since social problems such as crime and unemployment involve economic costs, social policies preventing or solving those problems can be reframed as economic investments that generate savings for the public budget. Another example concerns the provision of childcare services, which can be regarded as a double investment: they improve children’s human capital – with long-term returns in terms of growth and productivity – and they allow women to enter and stay in the labour market, thereby directly contributing to higher employment rates. Re-thinking social policies as economic investments, in turn, enables the development of ‘social impact bonds’, which are financial instruments that allow private actors to invest in social policy interventions earning a profit on them.

In a similar vein, within the context of the green growth agenda, nature is reframed in economic terms as ‘natural capital’, allowing to develop ‘green bonds’. The goal here is not only to make economic growth environment-friendly: the ecological transition itself should become a new engine of growth, a generator of jobs and an investment opportunity able to sustain profit-making through the creation of new markets (Kenis and Lievens, 2015). In particular, one of the main ways to reconcile social, ecological and economic objectives is to focus on creating ‘green jobs’. Increasing employment opportunities is considered the best strategy to secure inclusive growth and the fact that these new jobs are created in the ‘green economy’ allows to reconcile the social-economic and the ecological dimension. In this win-win-win scenario, social and environmental policies are re-framed in a way that makes them compatible – rather than in conflict – with economic growth.

To be sure, there are very different versions of public policies for the green transition. For example, Gabor and Braun (2025) propose a typology of ‘green macrofinancial regimes’. The today dominant approach involves ‘derisking’. In this regime, coordination operates via price signals, investment is guided by capitalists’ profit expectations and global market competition is the force that disciplines both private finance and states, encouraging them to invest in the green sectors and divest from carbon-based economic activities. In contrast, the regime involving a ‘big green state’ is based on state-led planning that steers (both private and public) investment and divestment, using carrots and sticks (for disciplining private capital) while also mobilizing directly sate-owned enterprises and public spending. While this regime is still marginal today (China is perhaps an exception), the crisis of neoliberal globalization – and the associated intensification of geopolitical tensions – as well as the deepening of the ecological crisis will possibly make this regime more and more attractive. Gabor and Braun (2025) propose a ‘democratic big green state’ as the ideal regime for promoting decarbonization in a just way. These arguments echo the growing interest in Green Keynesianism. In this context, some of the most progressive versions a ‘Green New Deal’ (e.g. Aronoff et al., 2019; Chomsky and Pollin, 2020; Pettifor, 2019) clearly oppose neoliberalism and in some cases even capitalism itself.

However, one limitation of many progressive proposals is that are centred on decarbonization – and thus on a radical shift to renewable energy – but this focus on the *sources* of energy does not interrogate the ways in which energy is *used*. In this way, many progressive approaches tend to neglect the need to downscale the production and consumption of non-essential goods and services in the global North (Schmelzer et al., 2022: 9-10). Moreover, the focus on decarbonization downplays the fact that also renewable energies depend on scarce resources – metals and minerals that are extracted mainly in the global South. As Ajl (2021) argues, Green New Deal proposals – even radical ones – usually neglect issues of anti-colonialism, global justice and climate debt. Finally, Green Keynesianism tends to promote the economization of social-ecological issues. Indeed, even the most progressive proposals of a Green New Deal either remain committed to economic growth (Mastini et al., 2021) or, when they reject growth, they remain ‘productivist’, framing the ecological transition as an opportunity to boost employment (Green, 2022).

### Common features

Despite their diversity, all these different initiatives tend to share three specific traits. The first common characteristic is the extension of the economic rationale to non-economic domains, which allows the commodification of areas of social-ecological life previously shielded from market logics. Thus, the discourses of ‘social investment’, ‘human capital’ and ‘natural capital’ spread the economic rationale to domains of ecological and social life that were previously largely uncolonized by this logic. Moreover, these discourses translate into concrete practices, such as ‘social impact bonds’ and ‘green bonds’; social-ecological labelling of products; and the systematic application of the cost-benefit logic to social and environmental policies. These practices tend to commodify social and environmental values such as justice (e.g. reduction of poverty and gender inequalities), human wellbeing (e.g. health) and sustainability (e.g. the protection of biodiversity), sometimes transforming them into products that ethically minded consumers and investors then buy on the market.

The second common aspect is that these initiatives tend to promote the de-politicization and de-democratization of social-ecological issues. On the one hand, the extension of the economic logic to all domains makes political opposition impossible (Brown, 2003). If everything – from social justice to environmental sustainability – can be framed from within the economic rationale, to which political values can oppositional forces refer to? Thus, democratic deliberation is superfluous and political choices become technocratic matters, with de-politicization presenting these choices as if they were inevitable, that is, motivated by objective, neutral and scientific facts (Kenis and Lievens, 2015). On the other hand, privatization and marketization empower private market actors (individual consumers; private companies; investors) at the expense of political-democratic subjects, such as parliaments, trade unions and civil society organizations. Hence, both CSR and ethical/political forms of consumption do not challenge the power of private capital: they actually contribute to consolidate and legitimizes this power, as they present capitalist corporations as the most appropriate actors to tackle social-ecological problems. Given the centrality of corporate power in neoliberalism (Hathaway, 2020), these processes reinforce a key feature of contemporary capitalism. Finally, commodification and de-politicization are linked to a third common element, namely the consensual, conflict-free approach that these initiatives entail (Kenis and Lievens, 2015). For example, with the rise of ethical/political consumption, the promotion of social-ecological justice simply requires shopping otherwise: there is no need to participate in strikes and demonstrations, to join a trade union, to regularly vote for political candidates or to engage in a social movement. Rather than civil society or the formal political sphere, it is the market that becomes the critical arena for addressing social-ecological problems – and the market notoriously involves consensus rather than conflict among the parties. The voluntary engagement of private actors within the market – rather than democratic politics – becomes the key for promoting social-ecological goals so that processes of economization/commodification and de-politicization/de-democratization sustain each other (Table 1).

**Table 1.** Towards social-ecological capitalism?

Social-ecological capitalism		
Level concerned	Example of initiative	Common processes
Individuals	Ethical-political consumption (e.g. buying fair-trade products and organic food)	Economization and commodification of ethical, political and environmental values De-politicization and de-democratization
Private companies	Corporate social responsibility (voluntary commitment to social-ecological standards)	
International organizations and national governments	Adoption of inclusive green growth principles in policymaking (e.g. creating ‘green jobs’ for promoting employment-rich decarbonization; investing in ‘human capital’ and ‘natural capital’; develop social and green bonds)	

## The economization paradox

These social-ecological versions of capitalism are characterized by a central paradox. Indeed, while they aim to promote social-ecological goals, they do so through the economization of these goals, that is, transforming them into economic goals. The paradox is that, within capitalist societies, higher degrees of economization are potentially associated with better social-ecological outcomes. For example, social investment is more generous than austerity (welfare retrenchment) *because* it reinforces both the epistemic and the distributive dimensions of capitalism (Laruffa, 2022). Hence, in all the cases discussed above, social-ecological problems are addressed without challenging capitalism in its epistemic and distributive dimensions and actually *reinforcing* them: not only the economic framework is extended to social-ecological goals (epistemic dimension) but when the latter are successfully transformed into commodities that can be exchanged in the market, they can become new sources of profits for the economic elites (distributive dimension). Promoting social-ecological goals *within* capitalism then requires to transform them into profitable investments. Thus, the *economization paradox* is that *higher* levels of economization allow comparatively *better* social-ecological outcomes. The economization of social-ecological goals allows to at least partially address the fundamental contradiction inherent to capitalism. Indeed, the latter tends to systematically undermine its non-economic background conditions of possibility: like a serpent that eats its own tale, ‘cannibal capitalism’ persistently devours the very supports (e.g. natural environment and social reproduction) on which it relies (Fraser, 2022). But the economization of social-ecological goals allows to integrate these non-economic conditions of possibility within the capitalist framework: assigning an economic value to them potentially allows to partially promote them *within* capitalism and its logics.

To be sure, the contradiction between a profit-driven economy and its non-economic social-ecological preconditions cannot be entirely resolved. Very often the contradictions are simply re-located in the future (through unsustainability) or in other regions of the world. Thus, the minerals and metals extracted in the Global South for the ‘green’ transition in the North in a neo-colonial manner clearly undermine both environmental sustainability and social justice in the South (Ajl, 2021; Bainton et al., 2021). Yet, in other cases, the contradiction can be effectively addressed within capitalism. Social policies aimed at solving various social problems with a view to reducing their economic costs make sense from *within* the capitalist logic of return on investment and cost-benefit analysis (Laruffa, 2022). In these cases, social reproduction is commodified, but the ‘crisis of care’ inherent to capitalism (Fraser, 2022: 53–74) is at least partially addressed. In the same vein, ethical and sustainable finance allows investing money in ‘responsible’ companies, thereby advancing social-ecological justice with respect to mainstream finance. As Hiss (2013) argues, despite its limitations, the ‘financialization of sustainability’ allows to make visible negative externalities that would be otherwise ignored, thereby brining sustainability into the decision-making processes of investors and corporations in a context marked by the absence of global regulations. Similarly, buying organic and fair-trade products surely contributes to promoting more just and sustainable modes of production. As Youde (2009) points out, while ethical forms of consumption do not amount to a ‘revolution’, they nevertheless pragmatically improve the situation within the current political economy structures.

Crucially, while economization may address social-ecological problems, it continues to undermine democratic governance, leaving power inequalities unchallenged and maintaining the economy out of democratic reach. The reason for this is twofold. On the one hand, the market cannot be democratized (Nullmeier, 2014). Market actors can be generous and act in conformity with ethical values (e.g. companies can engage in CSR and philanthropy; consumers can buy organic products), thereby promoting social-ecological goals. But voluntary exchanges between contractual parties cannot be democratized. On the other hand, the extension of the economic logic to non-economic domains de-politicizes issues, framing them in a technocratic manner that undermines the democratic quality of the public discourse and that impedes to deliberate on the nature of the good society (Sandel, 2012). In particular, the freedom to promote non-economic goals is severely constrained under capitalism: it seems that the only possibility to advance social-ecological aims is to reframe the latter in economic terms whereas societies cannot – on the basis of democratic deliberation and political choice – purse social-ecological goals that oppose the economic rationale or the demands of accumulation (Laruffa, 2022).

Thus, the paradox within capitalism can be formulated as follows: higher levels of economization and commodification – and thus of *depoliticization/de-democratization* – are potentially associated with better social-ecological outcomes. Within a capitalist system, initiatives relying on the economization and de-democratization of social-ecological goals have the potential to promote the latter, often representing an improvement with respect to the status quo. Expanding the share of organic agriculture and fair-trade products within mainstream capitalist economies surely involve an improvement with respect to an economy dominated by firms that do not embrace such social-ecological standards. Similarly, a policy that combines ‘social investment’ and ‘green-growth’ is clearly an improvement with respect to the continuation of fossil-fuel capitalism in combination with welfare retrenchment and an austerity-driven macroeconomic policy. Such social-ecological capitalism – if fully implemented – would be better than the varieties of capitalism that are currently hegemonic today. The paradox lies in the fact that these improvements are achieved through the economization of social-ecological values.

Figure 1 graphically summarizes the economization paradox. This graphic and those that will follow clearly do not do justice to the complexity of the real, reducing multidimensional phenomena such as social-ecological goals and de-democratization to simple curves. This way of displaying things may be even accused of reflecting precisely those ways of thinking that I am trying to problematize. Nevertheless, I think that these graphs help to visualize in a synthetic way the arguments of this paper, which is why I decided to keep them. Importantly, the economization paradox should be read mostly in hypothetical terms: while we can observe some of the described tendencies in reality, a fully social-ecological version of capitalism is far from being dominant today.

**Figure 1.**
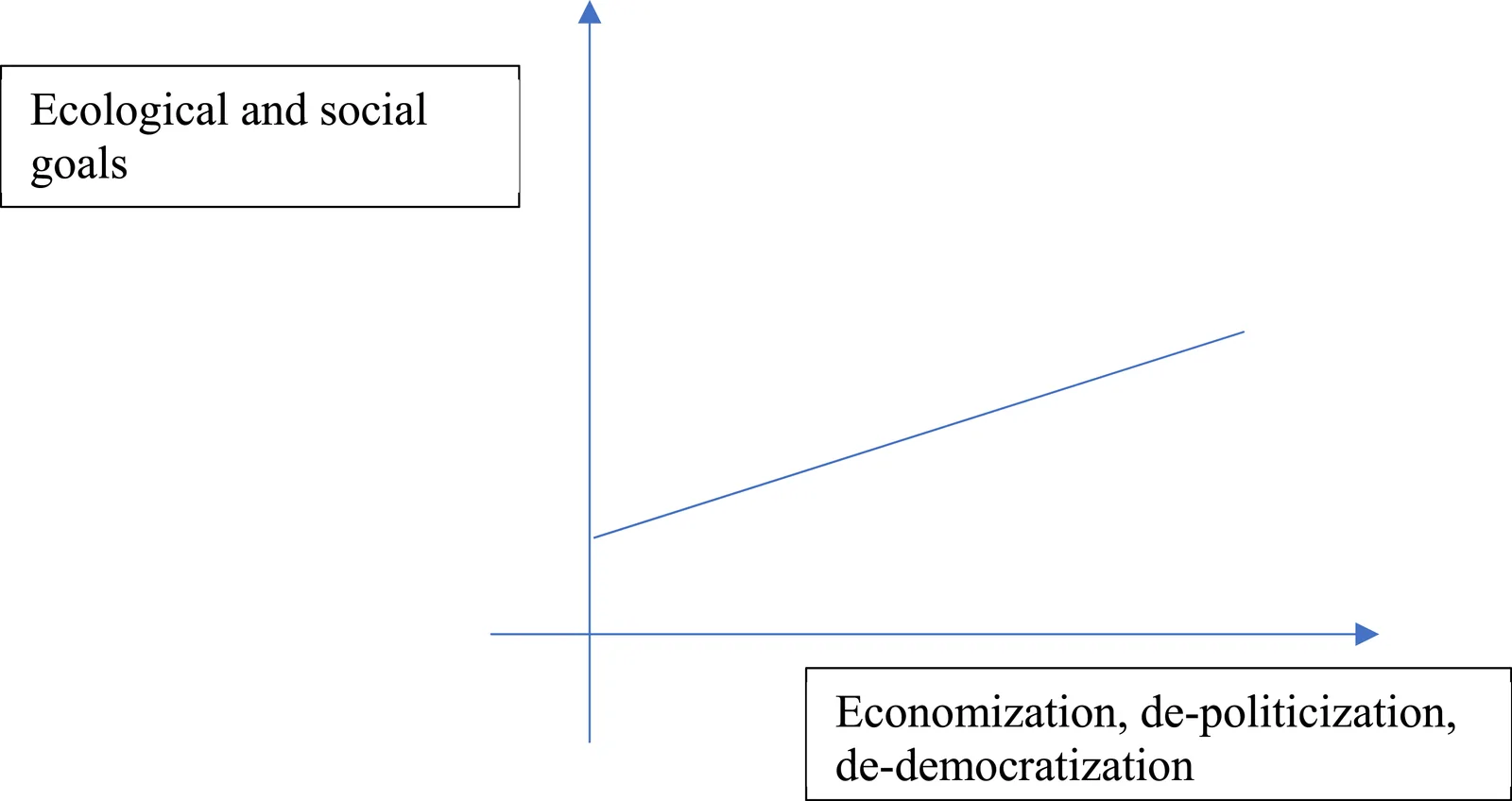
The economization paradox. Promoting social-ecological goals through economization and de-democratization.

## The progressive dilemma

The economization paradox generates a dilemma for those political forces that fight to develop progressive alternatives to capitalism. Since progressives struggle to address social-ecological problems in the current context, they may strategically adopt an economized discourse that highlights the economic benefits of promoting social-ecological goals. This is precisely what happened with social investment as a progressive welfare discourse: many supporters of this perspective pointed to the economic benefits of social policy for convincing political leaders and the economic elites to invest more money in social policy, ultimately with a view to ameliorating the living conditions of disadvantaged groups, such as homeless people and low-skilled unemployed (Laruffa, 2022). In the same vein, scientists and activists sincerely aiming at protecting nature often collaborate with mainstream economists with a view to developing models that make intelligible the value of nature to those powerful actors that think in quantitative and monetary terms: the strategy is to highlight the ways in which nature contributes to the economy (Dempsey 2016). While these attempts to protect society and nature through their economization often fail, they sometimes allow social-ecological criteria to be included in the decision-making of economic actors (Hiss, 2013). The dilemma hereby is that in order to promote social-ecological goals, progressive forces have to accept the capitalist framework, thereby further entrenching it, for example, through the application of the cost-benefit logic to non-economic domains and the commodification of previously uncommodified areas of social-ecological life.

An alternative political strategy would aim to realize the normative ideal of social-ecological justice, explicitly prioritizing social-environmental goals over economic ones (Grossmann, 2022). Such a strategy would consist in promoting a non-economized discourse, that is, moving beyond the ‘economic epistemologies’ of productivism (Green, 2022: 338). Thus, as Hermann (2021) argues, developing alternatives to capitalism requires to fight commodification, shifting the goal of the economy away from profits and market value toward democratically defined needs and use value. In contrast to improvements within capitalism, this alternative strategy would thus focus on the *subordination of the economy to the needs of society.* Democratization, in this perspective, requires *de-economization* in both epistemic and distributive terms. At the epistemic level, the economic rationale should be subordinated to a socio-political rationale. This clearly entails also a process of re-politicization of public issues that under capitalism are conceived as merely technical matters to be ‘solved’ and ‘managed’ through cost-benefit calculations. In this politicized approach, public policies are democratically discussed in terms of their impact on ‘final ends’ (such as human wellbeing and sustainability) rather than in terms of their consequences for economic growth. At the distributive level, ethical-political values are not commodified or transformed into profit-making opportunities. Rather, the goal of economic production shifts from generating private profits to the satisfaction of democratically defined social-ecological needs. This democratization of the economy is also unavoidably linked to processes of re-politicization: while the economization strategy reposes on a seemingly win-win logic that allows de-politicization and explicitly avoids open conflicts, the redistribution of political power in society entailed in the democratization strategy necessarily involves social-political struggles, as the dominant classes will not voluntarily give their power away as a generous concession. The key argument here is that for overcoming capitalism it is not enough to promote social-ecological goals: it is also essential to promote democratization processes aimed at redistributing power across society (Figure 2).

**Figure 2.**
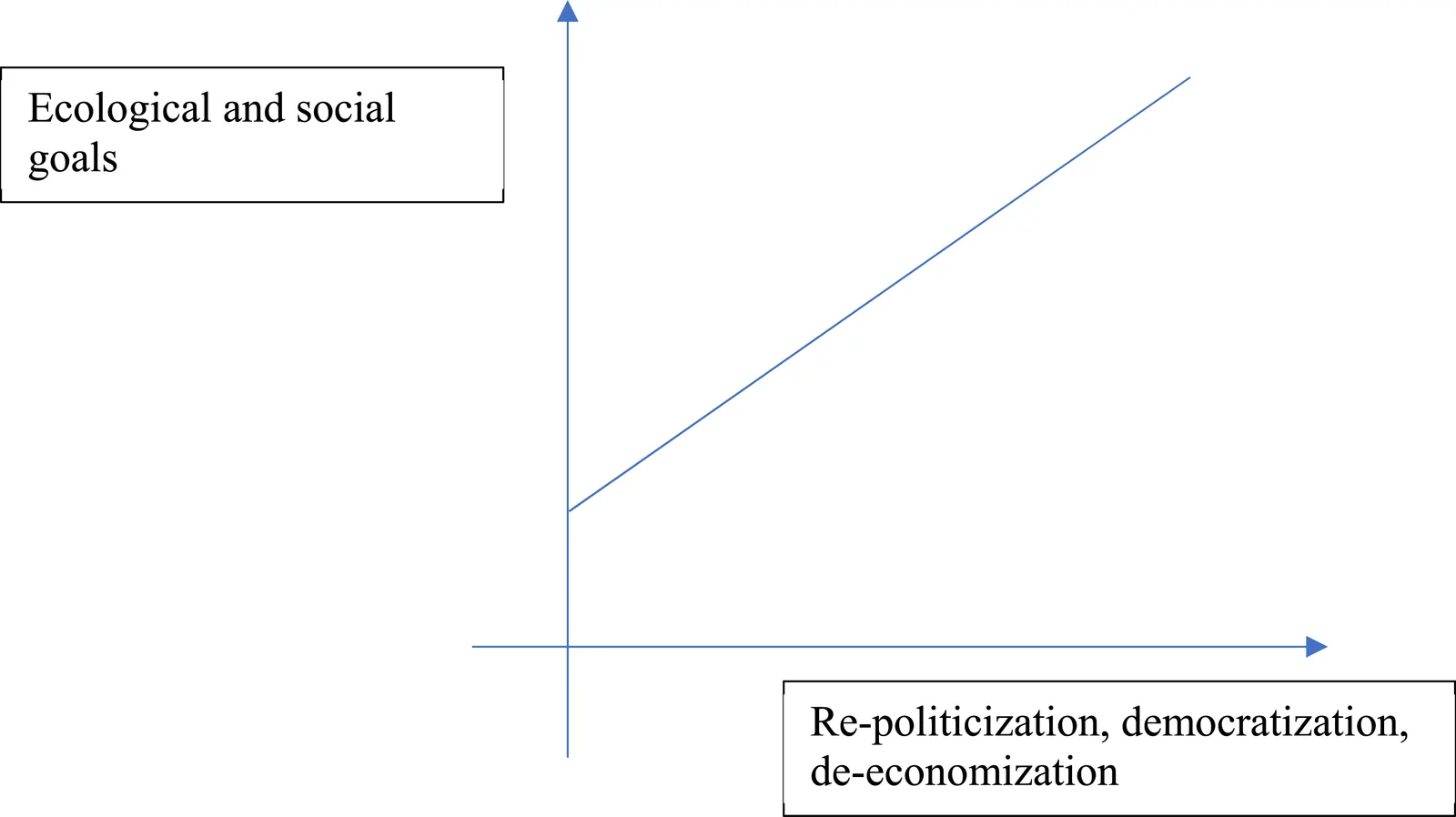
A progressive social-ecological alternative to capitalism: Promoting social and ecological goals through democratization.

Note that through processes of democratization it is possible to more effectively advance social-ecological goals than through economization: in Figure 2 the line reaches higher absolute levels and the slope of the line is steeper than in Figure 1. Indeed, the logic of profit-making tends to structurally narrow the possibility for social change. As Kenis and Lievens (2015: 49) argue, ‘capitalism can surely be corrected and reformed’, however, ‘it appears to be structurally incapable of the kind of holistic thinking that is necessary to understand ecology as such’. For example, in the field of green technologies, even when private entrepreneurs are genuinely motivated to ‘save the planet’ (i.e. going beyond a mere marketing strategy of greenwashing), human creativity is tamed by the pressure of commercial success, which compels entrepreneurs to focus on those marketable technologies that offer only incremental improvements to the status quo: those that involve slightly less wasteful and polluting production processes, more energy-efficient machines, biodegradable materials and cleaner energy sources (Goldstein 2018). Since innovations need to be funded, for-profit capital disciplines inventors’ creativity – which is effectively managed, controlled and ‘enclosed’ – thereby shaping the selective process through which some technologies are considered viable and funded whereas others are discarded and set aside. In this way, the market becomes the final arbiter of what futures are conceivable and desirable: ‘what is and is not possible’ depends on ‘what is and is not fundable’ (Goldstein, 2018: 93) and only technologies that provide incremental improvements in already established markets are funded. The innovative technologies that are funded are those that produce ‘non-disruptive disruptions’, providing ‘solutions’ in the current system but ‘without actually changing much of what causes the underlying problems in the first place’ (Goldstein, 2018: 10). Instead, technologies that have low potential in terms of growth and profits will not be funded – no matter how high their positive impact on ecological issues. From this perspective, the future is always ‘incrementally improved, but never fundamentally, transformed’ (Goldstein, 2018: 92).

At first sight, it seems that not only capitalism but also ‘democratization' will fail to enable a social-ecological transformation. The electoral victories of politicians that negate climate change and want to maintain fossil-fuelled capitalism (e.g. Trump) would be the proof for this claim. To a closer look, however, the problem is not with democracy itself but with the specific – very limited – form that democracy takes under capitalism, so that the solution is to reinforce the anti-capitalist ‘tendency’ of democracy (Hickel, 2022: 245–250). Indeed, members of the capitalist elites are those that (indirectly) detain political power – including the power to manipulate public opinion through (digital) media on which ‘fake news’ and ‘alternative facts’ proliferate. Contemporary democracies are closer to oligarchies than to real democracies. As Green (2022: 337) argues, corporations ‘have committed vast financial resources and political energy to the maintenance of an ecologically devastating status quo’ and any serious political project aimed at reconfiguring the political economy for the ecological transition has to confront the concentration of political and economic power through democratization.

However, the problem with an explicitly anti-capitalist strategy is that it may fail to influence the political agenda, becoming irrelevant in practice. Thus, the dilemmatic choice seems to be between accepting the capitalist logic with a view to promoting progressive goals within the contemporary system on the one hand, and refusing the capitalist framework, risking to be trapped in marginal political positions on the other. The economization paradox implies that promoting social-ecological goals in the current system may require a deeper embracement of its main features, whereas an approach based on values and norms opposing capitalism may ultimately fail to improve the situation.

## The non-linearity of progressive change between ‘unrealistic pragmatism’ and ‘necessary utopianism’

The progressive dilemma directly interrogates the relationship between ‘utopian’ normative principles and ‘pragmatic’ interventions. How can we pragmatically advance towards the ideal of social-ecological justice? While theories of justice often aim to describe perfectly just societies, Amartya Sen (2009) famously proposed a comparative approach to justice. The plurality of values, principles and worldviews – especially at the global level – makes it very difficult, if not impossible, to achieve a consensus on what constitutes a perfectly just society. But for Sen such an agreement is neither sufficient nor necessary for concretely advancing justice in the real world: it is not sufficient because agreeing on ideal institutions does not help us to choose among alternative effectively realizable options; it is not necessary because we do not need to agree on perfectly just institutions in order to start reducing injustice in the world. Adopting Sen’s comparative approach, social-ecological versions of capitalism would represent an advancement of social-ecological justice with respect to the status quo, which is often dominated by austerity and by highly environmentally unsustainable (carbon-based) and socially exclusionary (jobless) models of economic growth that benefits only a small minority on the top. However, social-ecological goals are promoted through the economization of the latter. This makes a post-capitalist world not entirely governed by the economic rationality and by profit-making more difficult, rather than easier, to achieve.

For example, expanding the market for fair-trade products would advance social justice in the world. However, the relative success of fair trade products should be understood in terms of its *compatibility* with capitalism: abandoning the early radical, anti-capitalist goals of the fair-trade *movement* – which aimed at making all trade fair through public regulation – fair-trade *labelling* involves a ‘market-friendly’ and ‘non-statist orientation’ focused on ‘voluntary commitments from corporations and consumers’ for addressing the most negative consequences of neoliberal globalization, without substantially challenging the latter – and giant profit-driven multinational corporations have now entered the fair-trade market, often becoming the dominant players within it (Fridell, 2007: 274–279). From this perspective, a central limitation of Sen’s comparative approach emerges from the fact that the path to justice may be non-linear. This implies not only accepting ‘the possibility that improvements in the long term requires some short-term sacrifice’, that is, that things have to go worse before that they can get better (Meshelski 2019: 34). I argue that non-linearity also means that comparatively improving the current situation may nevertheless entail a step in the ‘wrong’ direction, that is, a step that brings us more distant from normative ideals: improvements in the short-term may involve deteriorations in the long-term.

From the viewpoint of social justice, environmental protection, democracy and human wellbeing, a social-ecological transformation that abandons economic growth as a political goal is preferable to inclusive green growth (e.g. Sandberg et al., 2019). Yet, in the current context, the inclusive green growth agenda is easier to implement because it does not substantially challenge neither power inequalities nor the consumerist culture. Indeed, the inclusive green growth approach mainly entails technical solutions that do not require great changes in people’s behaviours and social practices or in the dominant culture. Nor do these technical solutions demand a strong redistribution of political and economic power: big companies would maintain their powerful and privileged position in society – these firms would only have to shift their strategies towards the production of fair-trade and organic products. In contrast, the post-productivist social-ecological transformation demands to abandon economic growth as a valuable political goal. This involves radical changes in people’ behaviours and values and a major redistribution of political power in society (including at the global level) and thus the inevitable emergence of conflicts – which clearly opposes the ‘win-win’ logic of inclusive green growth. As Buch-Hansen and Carstensen (2021) argue, while inclusive green growth involves a third-order paradigmatic change within the prevailing economic system, degrowth involves a fourth-order change, requiring to move beyond capitalism. Thus, the ecopolitical projects of degrowth and green growth are largely incommensurable: they substantially differ with respect to the magnitude of necessary changes; the central agents of change; the scientific knowledge at their bases; and in terms of their distributive consequences (Buch-Hansen and Carstensen, 2021). From this perspective, even if one agrees that degrowth would be in principle preferable to inclusive green growth, the former can still be discarded as utopian and unrealistic whereas the merit of the latter lies precisely in its political feasibility.

The point that I want to emphasize here is that the ‘pragmatic’ solution centred on inclusive green growth *reinforces* certain elements that the post-growth social-ecological transformation explicitly wants to challenge. In other words, the pragmatic solution does not constitute simply a realistic step in the direction of a deeper transformation: to some extent the pragmatic solution brings us in the *opposite* direction. For example, while a post-growth social-ecological transformation demands to de-prioritize economic production and employment, inclusive growth and Green Keynesianism reinforce the centrality of paid employment as the main gateway to social inclusion and participation in society through full employment policies (Green, 2022). In short, while the incremental/pragmatic approach centred on inclusive green growth comparatively improves the situation with respect to the status quo, it nevertheless implies a step in the ‘wrong’ direction with respect to the normative ideal: this paradigm reinforces central elements of capitalism – especially in terms of a deeper intrusion of the economic logic in social life (economization), with the consequent reinforcement of de-democratization tendencies (e.g. strengthening the power of corporations) – thereby undermining the possibility of fully realizing social-ecological justice in the long term. Figure 3 represents two hypothetical future scenarios. It shows how the social-ecological version of capitalism based on inclusive green growth (represented by the point B in the Figure) entails a political option that would *comparatively* improve the situation with respect to the status quo (point A). Yet, this economization-driven approach to the ecological transition would go in the *opposite direction* with respect to the ideal of a post-productivist social-ecological transformation aimed at the de-commodification of nature and society and at the democratization of social relations (point C).

**Figure 3.**
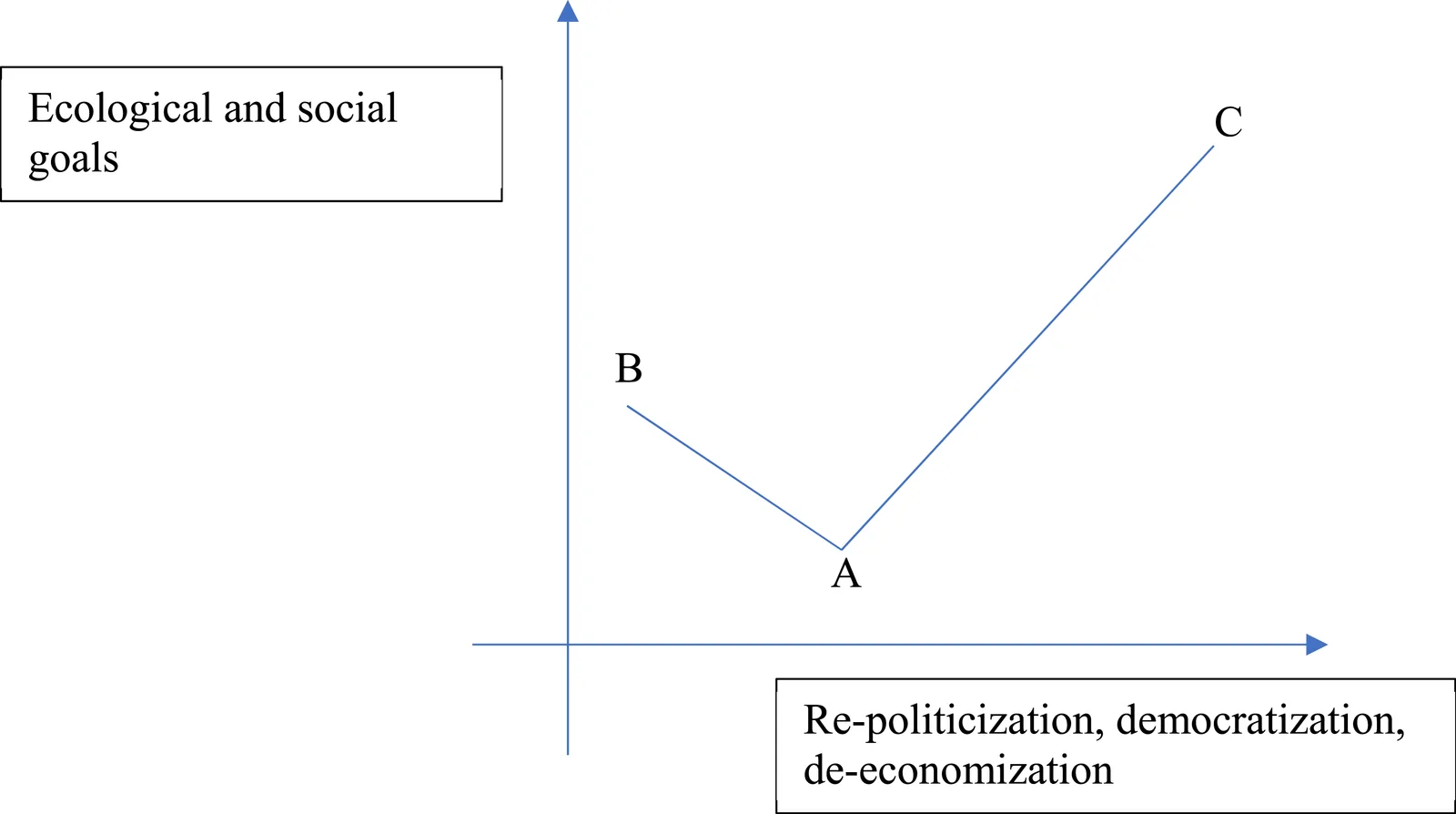
The problem of non-linearity in progressive social change.

Crucially, many disagreements concerning the pragmatic and the utopian positions are more linked to ‘epistemic’ rather than ‘normative’ divergences, as they refer to what actors consider ‘feasible’ – that is, what can be realistically changed and what instead must be accepted as given – rather than to different principles (Laruffa, 2025). When capitalism is considered to be without alternative, progressive efforts focus on comparatively improving the situation, even at the cost of reinforcing crucial elements of the current system. For example, many would in principle agree with Fraser (2022: 78) that capitalism ‘represents the sociohistorical driver of climate change, and hence the core institutionalized dynamic that must be dismantled in order to stop it’. Many would even embrace her normative vision of a democratic, ecological, anti-racist and feminist socialism, admitting that it represents a much more attractive way of organizing society than capitalism (Fraser, 2022: 141–157). Yet many would also argue that, in the present condition, it is impossible to overcome capitalism and that the socialist ideal is probably an unachievable utopia. Since we have to act now for improving justice and sustainability in the real world, we have to do compromises: we cannot wait for the end of capitalism to start promoting social-ecological goals. In this perspective, strategies relying on the economization of the latter (ethical-political consumption, CSR, inclusive-green growth) appear acceptable pragmatic solutions.

Given that anti-capitalism is currently nowhere on the political agenda, even overcoming capitalism demands pragmatic strategies that begin within capitalism. For example, Bärnthaler (2024: 4–6) emphasizes the importance of accepting compromises in the transition to a post-capitalist world: while the long-term goal remains that of overcoming capitalism, building ‘a hegemonic project here and now’ will ‘need to resonate with some capital fractions to be selected and retained’ so that ‘strategic overlaps with Green Keynesianism and social-democratic movements emerge’.

In contrast, other authors oppose this pragmatism. For example, Ajl (2021: 90) contends that the relationship between short-term ‘green Keynesianism and long-term eco-socialism is politically incoherent’. Thus, he argues against ‘splitting the antagonists’ camp, and getting certain sectors of capital on board’, claiming that this strategy forgets who pays the price of doing so: historically, social democracy ‘rested on profits from the Third World’ and current plans for green Keynesianism are equally ‘blind or uncaring about where value, capital, and profit come from’ (Ajl, 2021: 68–69). In this view, class compromises are bad also because they convince workers ‘to invest in the system, and to see their future as linked to its future’ (Ajl, 2021: 77). Finally, Ajl (2021: 165) observes that historical social democracy was ‘an achievement from below, not a grant from above’ and that it was the outcome of those ‘fighting for the abolition, not the softening, of capitalism’. From this perspective, even green social democracy – if achieved – will be the outcome of massive movements fighting for eco-socialism. In short, the problem of green social democracy is not only that ‘it is not achievable through current strategies’ and that, even if it were possible, ‘it would be imperialist’, resting on the exploitation of the South. The problem is also that ‘it limits our political imaginations’: while ‘to get anything anywhere close to eco-socialism, one must have a movement which is fighting for eco-socialism’, if ‘one is building a struggle that accepts a compromised horizon, one is not going to reach a further horizon’ (Ajl, 2021: 94–95).

In a similar vein, Green (2022) criticizes Green New Deal proposals that rely on productivist logics in the short-term for providing a capitalist stimulus in the green sectors, while hoping to break with capital and growth on the long-term. As Green (2022: 332) asks: ‘Can we move towards massive public investments, mobilizing a discourse of full green employment productivism (…), and then invert this logic, overnight, to something entirely opposite? How can the productivist framework and the full employment paradigm exist alongside a commitment to a steady-state economy and the reduction of production and consumption, that is, how can they enable a post-capitalist future? (Green, 2022: 334). According to Green (2022: 332), distinguishing between short-term and long-term strategies does not do enough to coherently reconcile these opposed political-economic logics.

While these dilemmas concerning whether a revolutionary or a reformist approach to transformation should be embraced are old as the Left itself, it seems that the ecological crisis makes these not only more urgent but also more radical. In this context, the problem is not only that what appears at first sight comparatively better may, in the long-run, be actually worse. The issue is also that what appears at first sight to be *realistic* may actually be unrealistic. The conventional view can be turned on its head: while it is probably impossible to dissociate economic growth – however ‘green’ – from environmental degradation (Hickel and Kallis, 2020), post-capitalist approaches discarded for being utopian may actually offer the only rational/possible solution to the contemporary crises. Indeed, while endless growth on a finite planet is illusionary, it *is* possible to subordinate the economy to the satisfaction of social-ecological needs. Post-capitalist approaches then are ‘utopian’ only in the sense that, instead of extrapolating the future from the present – accepting as given ‘the major contours of present society’ – they require thinking ‘first about where we want to be, and then about how we might get there’ (Levitas, 2001: 450). The dilemma that progressive forces face can thus be reframed as involving the choice between an *unrealistic pragmatism* – aimed at perpetuating capitalist growth, trying to make it green and inclusive – and the *necessary utopianism* of moving beyond growth-dependent capitalism.

Of course, it is a simplification to argue that there are only two options. The fact that we can observe in the world the presence of many different types of organizations (social enterprises, non-profit cooperatives, etc.) implies that it is pragmatically possible to start building social-ecological alternatives right now. Thus, some practices that already exist within capitalism, such as organic agriculture and the circular economy, may be part of a post-capitalist world. But the question remains: would the expansion of the circular economy and of the market for fair-trade and organic products within capitalism lead to a deeper social-ecological transformation beyond capitalism? While the arguments developed in this article do not provide a definitive answer, they at least suggest caution: there is no automatism in this process and progressive forces should be attentive to the pitfalls involved in it.

## Conclusion

In this paper, I discussed a central paradox that characterizes contemporary capitalism and the hypothetical future scenarios that could emerge in response to current multiple crises: higher levels of economization of social-ecological goals may be associated with an advancement of social-ecological justice. To be sure, fully realizing the latter is impossible within capitalism, that is, the social-ecological contradictions of capitalism cannot be resolved within this system. However, assigning an economic value to social-ecological goals may help to promote them as long as we are stuck in capitalism. Yet while the emergence of social-ecological versions of capitalism would comparatively improve the situation with respect to the status quo, they at the same time constitute a step in the ‘wrong’ direction – a step that, at least in certain aspects, brings the world in the opposite direction with respect to the normative ideal of social-ecological justice. Indeed, the paradox implies that promoting social-ecological goals *within* capitalism may require to further sustain economization-driven de-democratization, for example, the de-politicized nature of public discourse alongside the power of corporations and technocratic agencies. This problem of non-linearity in social change – which is largely ignored in comparative approaches to justice – generates a dilemma for progressive forces, as the risk of focussing progressive efforts on an explicitly anti-capitalist strategy centred on democratization and de-commodification is that of remaining trapped in marginal positions, ultimately failing to influence the political agenda. These difficulties are also linked to the tension between pragmatism and utopianism. Indeed, many disagreements among progressive actors do not derive from normative disputes at the level of principles, but from divergent ontological-epistemic positions, regarding what parts of social reality – especially capitalism – can be reformed and what instead should be pragmatically accepted as given (Laruffa, 2025). However, while at first sight those approaches that attempt to promote social-ecological goals within capitalism appear realistic, on the long-term they are actually unable to address the polycrisis. In contrast, while post-capitalist approaches are often discarded as naïvely utopian, they may actually offer the only rational solution to the crisis, namely abandoning the myth of endless growth, subordinating the economy to the satisfaction of democratically defined social-ecological needs. Thus, progressive forces seem trapped between an ‘unrealistic pragmatism’ and a ‘necessary utopianism’.

What are possible ways out of these progressive dilemmas? While there is no final answer to this question, solutions should probably focus on the idea of ‘revolutionary realpolitik’ proposed by Rosa Luxemburg. As Brie and Candeias (2019) argue, this idea indicates the need to implement realistic, achievable and concrete reforms in the current system that, however, pursue transformative goals, thereby overcoming the dichotomy between reform and revolution. While what appears unrealistic in daily politics is actually necessary for addressing capitalist contradictions, ‘system-hopping’ is impossible and ‘transformative steps’ are needed, as the risk of concentrating on a rupture is that of becoming political incapable of acting, condemning oneself to ‘revolutionary waiting’. In this perspective, reforms – no matter how pragmatic and incremental – should improve people’s living conditions in the present within the existing structure (also with a view to building political support), while at the same time opening the way for further transformation, facilitating radical change in the long-term.

Today, a revolutionary realpolitik should probably involve an alliance between productivist and post-productivist progressive approaches to the social-ecological transformation. Rather than searching a direct confrontation between these opposed paradigms, the first pragmatic reforms could be legitimized both within a productivist framework (e.g. because they create employment in ‘social’ and ‘green’ sectors) and within a post-productivist logic (e.g. because they prioritize use-value over exchange-value). In particular, investing in universal public goods may be such a pragmatic but transformative policy: it has the potential to ameliorate people’s quality of life while improving ecological outcomes, as public services are less intensive than their private equivalents (Hickel, 2022: 176–188). Extending de-commodified relationships, the expansion of welfare states and the provision of public goods could in turn reduce the demand for private goods and thus sustain the support for post-productivist policies aimed at downscaling unnecessary production and consumption (see also Bärnthaler, 2024).

Hence, in many cases, the progressive dilemma cannot be resolved at the level of principles, but it can be ‘avoided’ in practice. Progressive forces could thus try to adopt a double strategy that attempts to address social-ecological problems within capitalism while at the same time trying to overcome the latter, opening the way to a transformation and transcendence of this very system. Indeed, ‘eroding’ capitalism requires multiple strategies at different levels (Wright, 2021). For example, as a consumer one can buy fair-trade products while at the same time participating, as a citizen, in movements that struggle for making *all* trade fair (Jaffee, 2007: 266). In this context, progressive scholars have a role to play, too. First, representing the economy as heterogeneous is already an ‘ethical intervention’ that has the potential of ‘opening up the space of possibility’ highlighting the feasibility of non-capitalism in the here and now (Gibson-Graham and Dombroski, 2020: 19). Second, scholars-activists can work with anti-capitalist movements for creating pressure on governments from outside, being aware that the horizon of eco-socialism is necessary even for obtaining green social democracy (Ajl, 2021). Finally, progressive scholars are needed also for developing policy proposals in the context of ‘top-down governing’ (Bärnthaler, 2024: 8), making sure that pragmatic policies are as transformative as possible.
